# Single‐Cell Profiling Reveals Conserved Differentiation and Partial EMT Programs Orchestrating Ecosystem‐Level Antagonisms in Head and Neck Cancer

**DOI:** 10.1111/jcmm.70575

**Published:** 2025-05-03

**Authors:** Donghui Jiang, Xiaoguang Wu, Yuanyuan Deng, Xi Yang, Zhiqiang Wang, Yong Tang, Li He, Xiaoguang He

**Affiliations:** ^1^ Department of Otolaryngology & Head and Neck Surgery First Affiliated Hospital of Kunming Medical University Kunming Yunnan China; ^2^ Department of Dermatology First Affiliated Hospital of Kunming Medical University Kunming Yunnan China; ^3^ Department of Radiation Oncology First Affiliated Hospital of Kunming Medical University Kunming Yunnan China

**Keywords:** cNMF, HNSC, meta programs, single‐cell sequencing, TME

## Abstract

Head and neck squamous cell carcinoma (HNSC) exhibits profound intratumoral heterogeneity, driven by dynamic interactions between malignant cells and the tumour microenvironment (TME). Using consensus non‐negative matrix factorisation (cNMF) on multi‐site HNSC single‐cell transcriptomes, we resolving conserved meta‐programs define cellular ecosystems. Six major epithelial programmes emerged, including a differentiation‐associated programme (Epi_Diff) correlated with SPDEF activity and favourable patient prognosis, and an invasive programme (Epi_pEMT) potentially controlled by TEAD4‐mediated ECM remodelling, exhibiting partial EMT markers (VIM, TGFB1). Compartment‐specific crosstalk analysis revealed Epi_pEMT cells may coordinate with mCAF1 fibroblasts and TAM(SPP1) through COL1A1‐CD44 and SPP1‐CD44 signalling, suggesting potential formation of a pro‐invasive niche. Conversely, Epi_Diff cells may interact with NK/T cells through CEACAM5‐CD8A and CCL5‐ACKR2, and may contribute to inhibit immune infiltration. Multi‐compartment correlation analysis revealed three ecosystem‐level patterns: (1) Inverse association between Epi_Diff and Epi_pEMT (Spearman *R* = −0.43); (2) Negative correlation between mCAF1 abundance and cCAF frequency (*R* = −0.48); (3) TAM(SPP1) dominance inversely correlating with both TAM(C1Q) (*R* = −0.43) and NK/T infiltration (*R* = −0.36). These axes suggest a potential hierarchical ecology framework where lineage‐specific polarisation and inter‐compartment synergies may collectively govern disease progression.

## 1 Introduction

Head and neck squamous cell carcinoma (HNSC), a malignant tumour originating from the mucosal epithelium of multiple anatomical sites in the upper aerodigestive tract, accounts for approximately 3.6% of global cancer‐related mortality [1, 2]. HNSC exhibits high aggressiveness with a propensity for cervical lymph node metastasis [3]. Current clinical management primarily relies on radical surgical resection combined with adjuvant radiotherapy and/or chemotherapy [4, 5]. Although immune checkpoint inhibitors have demonstrated clinical promise [6, 7, 8, 9, 10], their therapeutic benefits remain restricted to specific patient subgroups, highlighting the critical need for predictive biomarkers to optimise treatment strategies.

Intratumoral heterogeneity (ITH), emerging from the dynamic interplay of genetic alterations, epigenetic modifications and microenvironmental cues, critically modulates therapeutic response and clinical progression in HNSC [11]. Recent advances in single‐cell RNA sequencing (scRNA‐seq) have delineated ITH‐associated transcriptional regulators, termed “meta‐programs” (MPs), which are coordinated gene expression programs across cancer cells that occur within and transcend individual tumour boundaries [12]. In the context of HNSC, four MPs have been molecularly defined: (a) cell cycle, (b) interferon response, (c) partial epithelial mesenchymal transition (pEMT) and (d) epithelial senescence/differentiation, where the pEMT and senescence/differentiation programs demonstrate clinical relevance to metastatic dissemination and therapeutic resistance [13, 14]. Intriguingly, pEMT cells exhibit an inverse correlation with epithelial senescence/differentiation markers. pEMT drives invasion and metastasis via interactions with CAFs, remodelling the tumour microenvironment and enhancing tumour aggressiveness [13].

However, the intricate relationships the association between malignant MPs and tumour microenvironment cells remain poorly understood. Studies have found causal links between cancer cell states and specific cell types within the tumour microenvironment (TME) [15]. The interactions among these cellular components are crucial to tumour progression [16]. Thus, we employ an integrative computational approach to delineate interactions between HNSC cellular states and specific TME components, with particular focus on epithelial senescence/differentiation and pEMT programmes. Through application of the cNMF algorithm to parse scRNA‐seq data, we seek to uncover key biological mechanisms and potential therapeutic targets that could improve clinical outcomes for HNSC patients. Our findings may contribute to a deeper understanding of the intrinsic biological characteristics and offer new insights for tumour ecosystem therapy [17].

## 2 Materials and Methods

### 2.1 Processing of HNSC Tumour Tissue Samples and Library Construction

Following approval from the Ethics Committee of the First Affiliated Hospital of Kunming Medical University (Ethics Approval No. 2023L76), we collected fresh surgical specimens from the primary tumours of six patients diagnosed with laryngeal squamous cell carcinoma (LSCC). All participants provided informed consent without receiving any compensation, and the study was conducted in compliance with all relevant ethical guidelines.

Fresh LSCC tissue samples were obtained from primary tumour sites during surgery. The specimens were washed with phosphate‐buffered saline (PBS, Gibco) to eliminate residual blood and carefully trimmed to exclude adjacent adipose and fascial tissues. Tissues were then chopped into smaller pieces and dissociated using a Human Tumour Dissociation Kit following the manufacturer's guidelines, with Red Blood Cell Lysis Buffer (Miltenyi) applied to remove erythrocytes. Cell viability was evaluated via Countstar Automated Cell Counter, confirming that viability rates exceeded 85%. The dissociated cell suspensions were filtered through a 100 μm SmartStrainer and centrifuged at 400 × *g* for 5 min at 4°C. Cell pellets were collected and processed using the Chromium Next GEM Single Cell 5′ Library and Gel Bead Kit V2 (10x Genomics) according to the manufacturer's protocol. The resulting cDNA libraries were sequenced on an Illumina NovaSeq 6000 platform. FASTQ files were aligned to the human genome (GRCh38) using Cell Ranger software (version 6.1.2) for subsequent analyses.

### 2.2 Data Processing

We performed initial data processing using Scanpy (v1.9.6) for size correction and logarithmic normalisation, and the resulting count matrices were converted into a Seurat object by the ‘Seurat’ R package (version 4.0.2). We combined six laryngeal carcinoma samples from our study with publicly available data (GSE164690), which comprises tumour samples containing both CD45‐positive and CD45‐negative cell populations. Quality control criteria were applied to retain cells with over 500 detected genes, UMI counts between 500 and 10,000, mitochondrial gene content under 20%, and haemoglobin gene content below 1%. Batch effects were mitigated using Harmony (v1.2.3) with default parameters. SCTransform (v0.4.1) was utilised for normalisation and standardisation. For dimensionality reduction, we employed Principal Component Analysis (PCA) and Uniform Manifold Approximation and Projection (UMAP) with default parameters, followed by clustering to identify different cell populations. Clustering was performed using the Louvain algorithm, with the resolution parameter selected from a range of 0.2 to 1.0 to best identify distinct cell populations.

### 2.3 Cell Type Identification

Differentially expressed genes (DEGs) for each cell cluster were identified using Seurat's ‘FindAllMarkers’ with default settings and Wilcoxon tests. Upregulated genes guided cell type assignments based on established marker expressions. Annotations were assigned using established marker genes from HNSC studies in combination with the SingleR package (v2.0.0) [18]. Subpopulations were further resolved through additional normalisation, dimensionality reduction and clustering.

### 2.4 Identification of Malignant Epithelial Cells

To differentiate malignant from non‐malignant epithelial cells, we employed inferCNV to analyse the expression matrices of epithelial cells using default parameters, with non‐epithelial cells (immune cells and fibroblasts) serving as the reference group. CNV scores were calculated for each epithelial cell to quantify genomic deviations from normal states. Malignant cells were identified based on their CNV scores and correlation patterns [19]. We repeated the normalisation, dimensionality reduction and clustering procedures to refine the classification of malignant cell populations.

### 2.5 Analysis of Intratumoral Expression Programs and Meta‐Programs

To capture intratumoral heterogeneity, we excluded samples with fewer than 100 cells and applied consensus non‐negative matrix factorisation (cNMF) to expression matrices. Data were centred, and negative values were set to zero. Optimal K and recurrent programmes were selected based on > 50% gene overlap within tumours, ≥ 20% sharing across tumours and functional similarity using clusterProfiler 4.0 [20]. The identified expression programmes clustered into meta‐programmes via Pearson correlation. Top 50 genes per meta‐programme were averaged for signatures. UCell scored MP expression per cell, identifying high‐score MP cells. A similar approach was used for non‐malignant cells.

### 2.6 Differential Expression Analysis

Differentially expressed genes (DEGs) were identified using Seurat's ‘FindMarkers’ function. The Wilcoxon rank‐sum test was applied, selecting genes with adjusted *p*‐values below 0.05 and average log_2_ fold change greater than 1.2. Genes were further filtered by an expression difference threshold (d > 0.4) for subsequent analyses.

### 2.7 Functional Enrichment Analysis

We generated heatmaps of differentially expressed genes across cell subpopulations using the FeatureHeatmap function from the SCP package (v0.5.6). Subsequently, gene set enrichment analysis was performed on DEGs, utilising Gene Ontology (GO) terms and KEGG pathways for functional annotation.

### 2.8 Single‐Sample Enrichment Analysis

We obtained Hallmark gene sets from MSigDB [21] and metabolic gene sets [22], selecting pathways linked to cancer progression. Single‐sample gene set enrichment analysis (ssGSEA) was performed using the GSVA package [23].

### 2.9 Inferring Gene Regulatory Networks Using SCENIC

We employed SCENIC to infer gene regulatory networks, retaining genes expressed in over 3% of cells. The normalised expression matrix and cell metadata from Seurat were converted to loom files. Using pySCENIC (v0.10.2), GRNBoost2 inferred regulatory networks, followed by motif enrichment with RcisTarget to identify regulons and transcription factor (TF)‐target interactions. AUCell assessed regulon activity, generating an activity matrix to identify TFs with cell type‐specific activity. The top five TFs had their target genes ranked by regulation score, selecting the top 20 to construct regulatory networks, visualised with the ggraph package.

### 2.10 Cell–Cell Communication Analysis

We employed the CellChat R package and CellPhoneDB (v5.0) to examine interactions between malignant and non‐malignant cells by evaluating ligand‐receptor pairs across all cell types. The top 100 ligand‐receptor pairs, based on weighted average interactions and *p*‐values < 0.01, were selected and quantified. Interaction strengths and significance were visualised using dot plots and heatmaps generated with ggplot2.

### 2.11 Cell Potential and Developmental Trajectory Inference

Differentiation potential was assessed using CytoTRACE (v0.3.3) [24]. Pseudotime analysis and cell differentiation trajectories were inferred with the Monocle package (v2.0) using default parameters.

### 2.12 Statistical Analysis

All bioinformatics analyses were performed using R (v4.3.0) and Python (v3.7.16). The relative proportion of cell types in the TCGA_HNSC dataset was estimated using signature genes from scRNA‐seq data and the CIBERSORTx online tool (https://cibersortx.stanford.edu/). Enrichment scores were computed via GSVA (v1.38.2) using the gsva function.

Differences in cell type proportions across clinical stages were analysed using Kruskal‐Wallis tests (*α* = 0.05). Significant results (*p* < 0.05) underwent Dunn post hoc tests with FDR correction (*α* = 0.05). Patients were stratified based on median or optimal cutpoints determined by surv_cutpoint. Survival differences were assessed using Kaplan–Meier curves and log‐rank tests (*p* < 0.05).

## 3 Results

### 3.1 Single‐Cell Profiling Reveals Cellular Composition and Dynamics in HNSC

**FIGURE 1:**
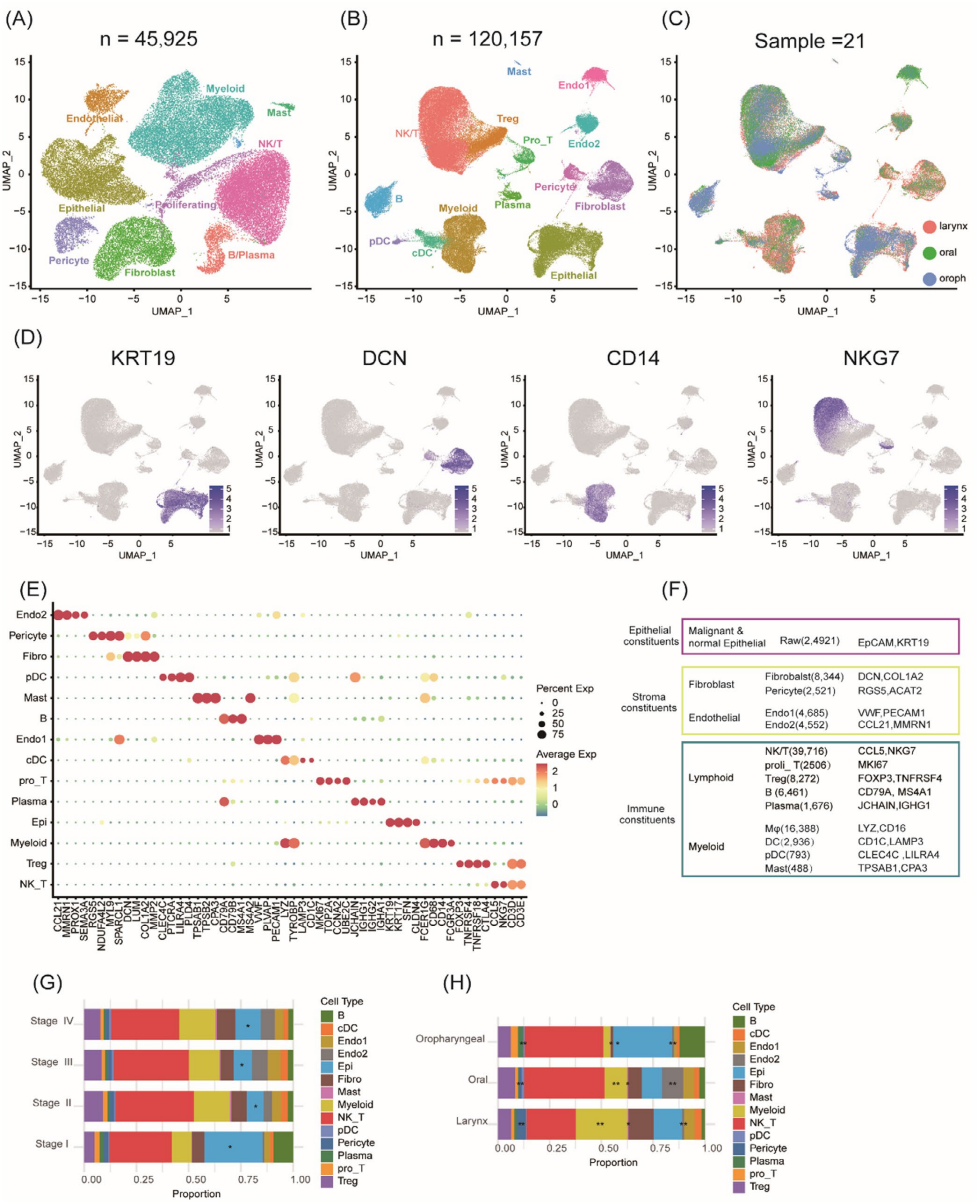
Overview of single‐cell transcriptomic profiling in HNSC patients. (A) UMAP visualisation of TME cellular composition using scRNA‐seq in six untreated laryngeal cancer patients across glottic, supraglottic and transglottic subsites. (B) UMAP visualisation demonstrates scRNA‐seq integration of 6 untreated laryngeal carcinoma samples with GSE164690 cohort. (C) UMAP analysis displaying the distribution of cells from seven laryngeal, eight oral and six oropharyngeal cancer patients. (D) UMAP visualisation of individual cell clusters annotated based on canonical lineage markers. (E) The dot plot shows the main markers of the primary cell types. (F) Summary of the number and markers of lineage cells. (G, H) The stage‐resolved and anatomically stratified cellular composition heterogeneity in HNSC is demonstrated through annotated cell‐type proportions (Kruskal–Wallis; *p < 0.05, **p < 0.01).

To explore interactions and dynamics between malignant cells and the tumour microenvironment (TME) in HNSC, we collected fresh samples from six untreated patients with glottic, supraglottic and transglottic cancers. Single‐cell RNA sequencing (scRNA‐seq) was conducted using the 10× Genomics platform (Figure 1A, Figure S1A). We obtained scRNA‐seq profiles of 45,925 cells with cell counts per patient ranging from 6686 to 10,210 (Figure 1A, Figure S1 and Table S1A).

Data integration boosts statistical robustness, mitigates heterogeneity and batch effects and facilitates generalisable insights. By integrating dataset GSE164690 [16], under stringent data quality control (Figure S2) and filtering, batch effects were eliminated using the Harmony algorithm. The QC and filtration steps yielded a total of 120,157 cells, which were displayed with UMAP (Figure 1B). Pathological verification was performed for all samples, with demographic, clinical details and quality control metrics provided in Table S1. We analysed samples from seven laryngeal, eight oral and six oropharyngeal cancer patients (Figure 1C), encompassing tumour stages I–IV. Based on the expression of classical lineage markers and functional enrichment analyses (Figure 1D,E; Figure S3), we identified three major cellular compartments—epithelial cells, stromal cells and immune cells—categorised into 14 distinct cell types, with specific subtypes and corresponding markers detailed in Figure 1F. Analysis of 21 samples across three anatomical sites and four disease stages demonstrated observable heterogeneity in cellular composition and infiltration patterns (Figure 1G,H).

### 3.2 Identification of Consensus Programs Within the Malignant Epithelial Cell

**FIGURE 2:**
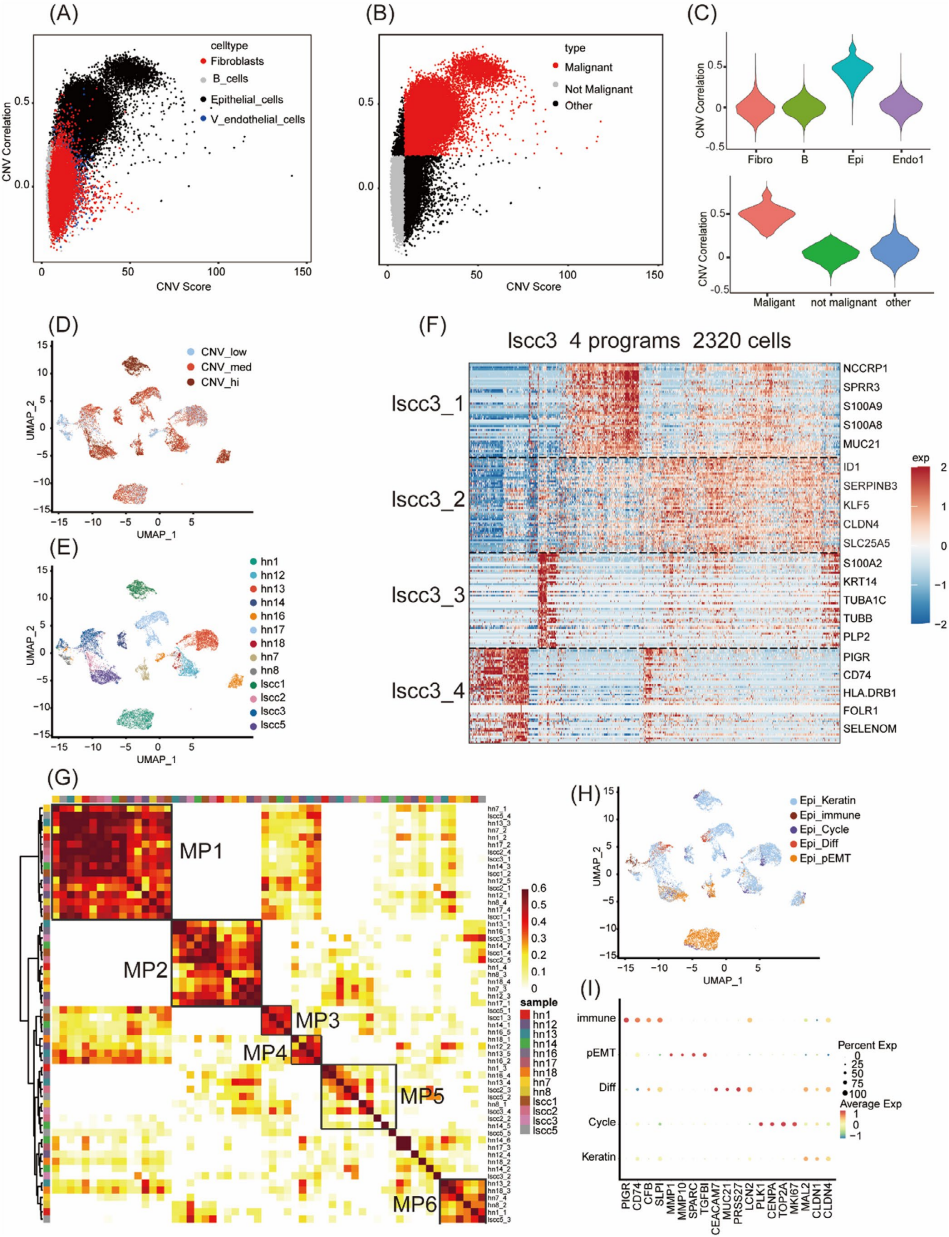
Characterising consistent malignant epithelial cell expression programs. (A, B) Heatmaps showing inferCNV analysis of CNV correlations between malignant epithelial cells and reference stromal and lymphoid cells, establishing a CNV correlation threshold to distinguish malignant from non‐malignant populations. (C) Violin plot depicting the distribution of CNV correlation coefficients among epithelial and reference cell populations. (D) UMAP projection illustrating the quartile‐based distribution of CNV intensity in malignant epithelial cells, highlighting variability in CNV profiles. (E) UMAP with dendrogram demonstrating that malignant epithelial cells cluster predominantly according to patient origin. (F) Heatmap presenting cNMF analysis of expression programmes in the lscc3 sample. (G) Heatmap displaying hierarchical clustering of integrated malignant epithelial cell expression programmes across all patient samples. (H) UMAP projection of malignant cells scored using marker genes of specific metaprograms, revealing differentiated expression profiles. (I) Dot plot highlighting differentially expressed genes in cells with high scores for specific metaprograms.

We first isolated the epithelial‐like cell population using lineage‐specific markers, followed by filtering to exclude samples with fewer than 100 cells, remaining 13 samples. CNV scores for epithelial, stromal and lymphoid cells were computed using InferCNV (Figure S4), with stromal and lymphoid cells serving as references to detect malignancy‐associated populations (Figure 2A–D). This analysis identified 21,123 high‐confidence malignant epithelial cells from 24,921 raw epithelial‐like cells. Subsequent reclustering via Seurat revealed that most epithelial cells displayed malignant CNV profiles, forming patient‐specific clusters (Figure 2E). These clusters may reflect intrinsic biological variability between patients.

To systematically dissect intratumoral heterogeneity following CNV‐based malignant cell identification, we employed cNMF to analyse expression variability. Expression programs from individual patients served as statistical units for hierarchical clustering (Figure 2F and Figure S5), allowing identification of shared expression programs across at least three cases. This analysis revealed six major co‐expression groups spanning different tumours. A consensus ‘meta‐program’ was defined for each group (MP1‐6), scoring and annotating all malignant cells accordingly (Figure 2G).

Among the identified meta‐programs, MP1 emerged as the most prominent, clustering consistently across cases. Cells with high MP1 scores were enriched in Gene Ontology Biological Process terms related to keratinocyte and epidermal cell differentiation pathways but lacked senescence markers [14]. Thus, we termed this the epithelial cell differentiation‐related program (Epi_Diff), as shown in Figure 2H,I, and Figure S6A. This closely aligns with the ‘epi_diff1’ feature from Puram et al. [13] and the ‘iCMS3’ signature from Dai et al. [25], as shown in Figure S7B,C. Marker genes include those involved in inflammation and immune regulation (S100 family, IL1RN, LCN2 and ANXA1), cell adhesion and invasion (CEACAM family, LGALS3, ADGRF1 and SPRR) and metabolic reprogramming (DHRS9, CLIC3 and PSCA). Enrichment analyses using HALLMARK [21] and metabolic gene sets [22] revealed signatures of metabolic reprogramming and immune evasion, particularly in heme, pyruvate and tryptophan metabolism (Figure S6). The Epi_Diff program was the most prevalent, detected in 13 tumours, indicating its reproducibility and conservation across squamous cell carcinoma, potentially influencing tumour development, progression and therapeutic response [26, 27, 28].

MP6 is marked by high expression of EMT‐related genes (VIM and TGFB1), metabolic genes (LDHA and PKM), cytoskeletal genes (ACTB and FLNA) and immune regulators (LGALS1 and TNFRSF12A), but lacks classical EMT transcription factors and collagen genes, led us to define it as partial epithelial–mesenchymal transition (Epi_pEMT), as shown in Figure 2H,I, and Figure S6. Epi_pEMT regulates cell adhesion, polarity loss and migration, with immune involvement indicated by Allograft Rejection and Inflammatory Response pathways. Angiogenesis and Hypoxia pathways reflect tumour adaptation to hypoxia via LDHA, while WNT/β‐catenin, Hedgehog and Notch enrichment highlights its role in cell fate and proliferation, aligning with ‘epi_pEMT’ and ‘iCMS1’ features from Puram and Dai [13, 25], as shown in Figure S7B,C.

Among the other programmes, Epi_Cycle's high‐scoring marker genes are MKI67 and TOP2A, exhibiting evident G2M checkpoint and DNA synthesis features; Epi_Kera mainly expresses keratin‐related genes KRT6A and KRT19; Epi_Imm is enriched in key molecules closely associated with adaptive anti‐tumour immunity, including PIGR, CFB, SLPI, CD74 and HLA‐DR. Epi_Stress expresses cell stress‐related genes JUN and FOS. A detailed list of the top 50 marker genes for the malignant cell meta‐programs identified in this study and previously reported is provided in Table S2.

### 3.3 Deciphering the Potential Molecular Basis Driving Consensus Cancer Cell States

We applied single‐cell regulatory network inference and clustering (SCENIC) [29] to investigate potential transcriptional drivers of malignant cell states. Each state exhibited a set of characteristic transcription factors, as shown in Table S3.

**FIGURE 3:**
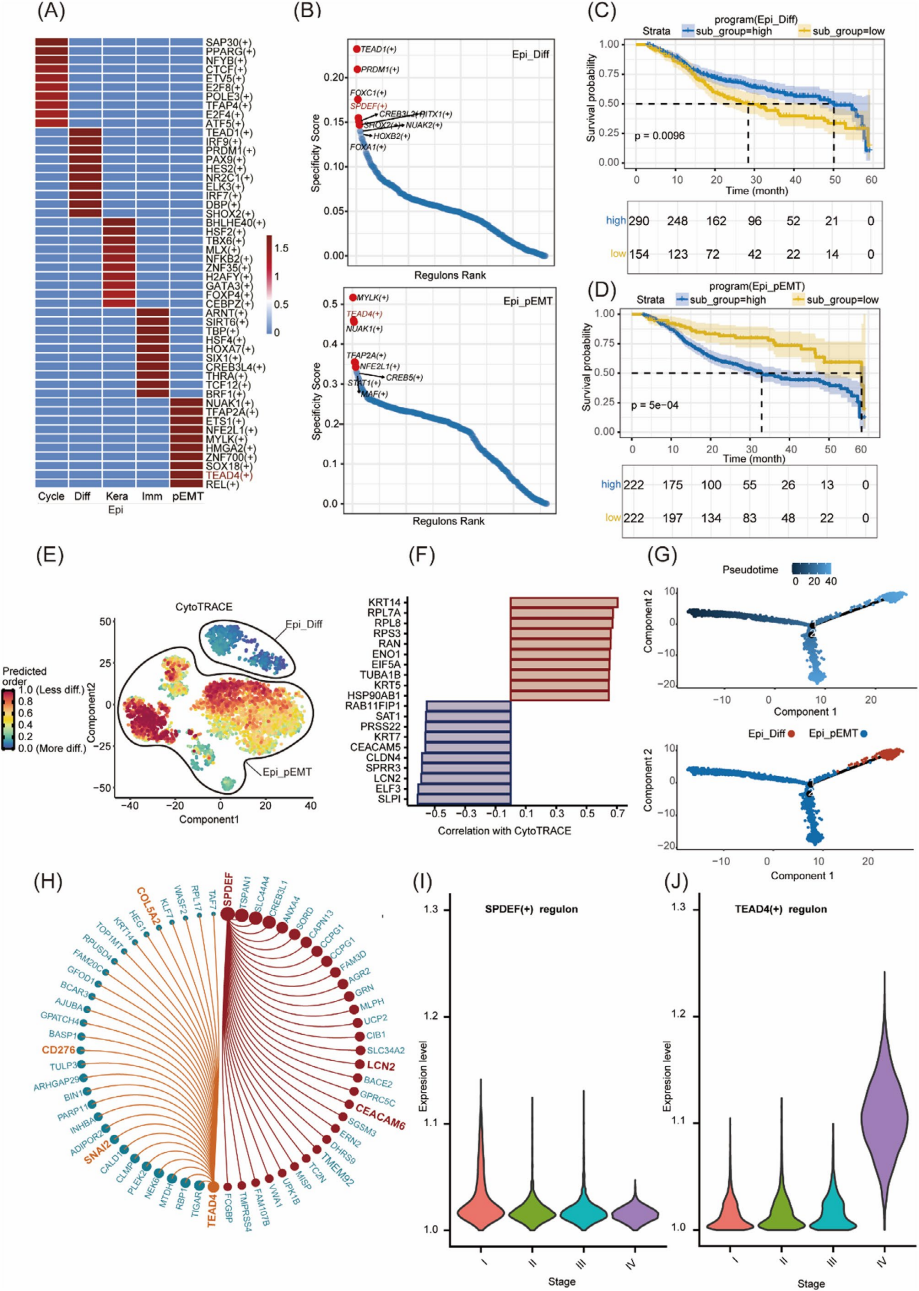
Unravelling the potential molecular mechanisms underlying consensus cancer cell states and their association with clinical prognosis. (A) Heatmap illustrating the top 10 highly expressed transcriptional regulators identified by SCENIC analysis across five distinct malignant epithelial cell states. (B) Dot plot displaying the top 10 uniquely activated regulators specific to the Epi‐Diff and Epi_pEMT cell states. (C) Kaplan–Meier survival analysis of the TCGA_HNSC dataset stratified by ssGSVA‐derived proportions of Epi‐Diff marker‐high cells. (D) Kaplan–Meier overall survival analysis comparing patients with high versus low expression of Epi_pEMT markers. (E) UMAP projection showing the predicted differentiation potential of Epi‐Diff and Epi_pEMT cells using CytoTRACE. (F) Bar plot of genes correlated with differentiation potential scores. (G) Integrated pseudotime trajectory analysis of Epi‐Diff and Epi_pEMT cells performed using Monocle2. (H) Circos plot from SCENIC analysis highlighting SPDEF and TEAD4 as potential regulators of target genes. (I, J) Violin plots illustrating the expression trends of SPDEF and TEAD4 across different clinical stages.

In the Epi_Diff state, we observed heightened activity of TEAD1, PRDM1, FOXC1, SPDEF and FOXA1. Notably, SPDEF is believed to play a crucial role in maintaining epithelial homeostasis and orchestrating differentiation programmes across multiple tissues. It may also exhibit tumour‐suppressive properties by promoting cellular quiescence [30]. In contrast, the Epi_pEMT state was characterised by elevated activity of MYLK, TEAD4, NUAK1, TFAP2A and NFE2L1. Among these, TEAD4 may regulate mesenchymal signatures and extracellular matrix remodelling through coactivator‐associated transcriptional programmes [31](Figure 3A,B).

Through TF‐target network reconstruction, SPDEF was computationally linked to Epi_Diff markers LCN2 and CEACAM6, aligning with its established role in mucin regulation [32] (Figure 3H). Notably, while canonical EMT markers were absent in Epi_pEMT cells, TEAD4 network modelling may have suggested its potential coordination of SNAI2 and CD276 expression (Figure 3H). Notably, the expression of SPDEF and TEAD4 showed opposing trends in IV‐stage tumours (Figure 3I,J).

We also assessed the prognostic value and differentiation potential of these cell states. Compared to Epi_EMT, cells with high Epi_Diff marker expression were associated with better clinical outcomes (Figure 3C,D). Additionally, CytoTrace and Monocle analyses confirmed that Epi_Diff cells predominantly correspond to terminally differentiated states (Figure 3E–G).

### 3.4 CAFs Shape Consensus Malignant Cell States via Specific Intercellular Communication

**FIGURE 4:**
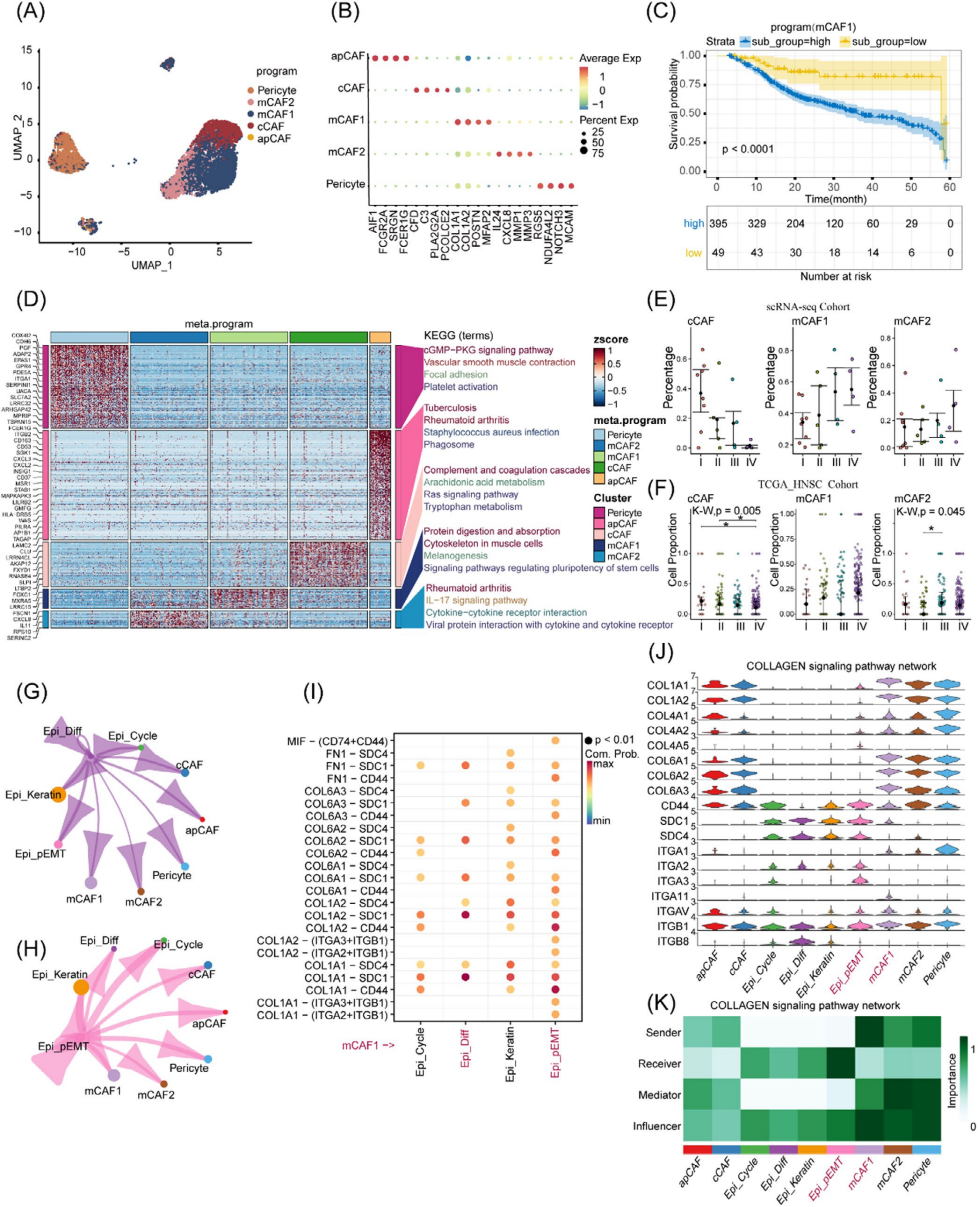
Analysis of the interactions between CAFs and malignant cell states. (A) UMAP dimensionality reduction plot displaying the clustering analysis of cancer‐associated fibroblasts (CAFs). (B) Dot plot illustrating the expression of top four signature genes specific to CAFs. (C) Deconvolution analysis of the prognostic features of CAFs within the TCGA_HNSC dataset. (D) Bar chart presenting the KEGG enrichment analysis of differentially expressed genes in CAFs. (E, F) Dot plot showing the relative proportions of CAFs in this single‐cell dataset and the TCGA_HNSC dataset. *p.adj value < 0.05 (Dunn's test with multiple comparisons). (G, H) Inferred ligand‐receptor interaction strength between Epi‐Diff or Epi_pEMT cells and CAFs, respectively. (I) The bubble plot of the ligand‐receptor pairs where mCAF1 acts as a sender to malignant cells. (J) The violin plot of the expression levels of ligand‐receptor genes across cell types in the COLLAGEN signalling network. (K) Highlighting the importance of each cell type in the COLLAGEN signalling.

We extracted fibroblast‐like cells using lineage‐specific markers and identified seven CAF‐related expression programs that corresponded closely to the cell type classifications (Figure 4A and Figure S8A). Through the utilisation of cell marker identification, differential gene enrichment analysis and correlation with clinical prognosis, we confirmed the cellular identities. mCAF1(COL1A1, POSTN) expression program is closely associated with the synthesis of the extracellular matrix (ECM), tissue remodelling and stromal support (Figure 4D). mCAF2 (CXCL8, IL24) is related to immune regulation, inflammatory response and matrix degradation, and may represent an immune/inflammatory regulatory phenotype of CAFs. The Pericyte (RGS5, MCAM) expression program is closely linked to angiogenesis and vascular stability. The cCAF (C3, CFD) expression program exhibits highly active complement system features, suggesting a potential role in complement activation and immune regulation. The apCAF (TYROB, HLA‐DRB1) expression program shows characteristics of antigen presentation and immune regulation. By performing deconvolution analysis on the TCGA_HNSC dataset, consistent with previous studies [33, 34], we examined the proportional distributions of cCAF, mCAF1, and mCAF2 across different clinical stages (I–IV) and assessed the relationship between their expression programmes and patient prognosis. The mCAF1 programme exhibited an increasing trend across stages I–IV, though this did not reach statistical significance. Prognostic analysis revealed that mCAF1 is associated with poorer overall survival (Figure 4C). cCAF decreased as tumours progressed, with significant differences between I versus IV and II versus IV stages (*p*.adj = 0.048) (Figure 4E,F).

Subsequent analyses focused on the interaction between malignant cells and CAFs. Comparative analysis revealed enhanced interaction potential between Epi_pEMT and mCAF1 relative to Epi_Diff, with notable enrichment of key ligand‐receptor pairs, particularly involving COL1A1‐CD44 and MIF‐CD74+CD44 (Figure 4F–H).

### 3.5 Myeloid Cell Crosstalk Malignant Cell

**FIGURE 5:**
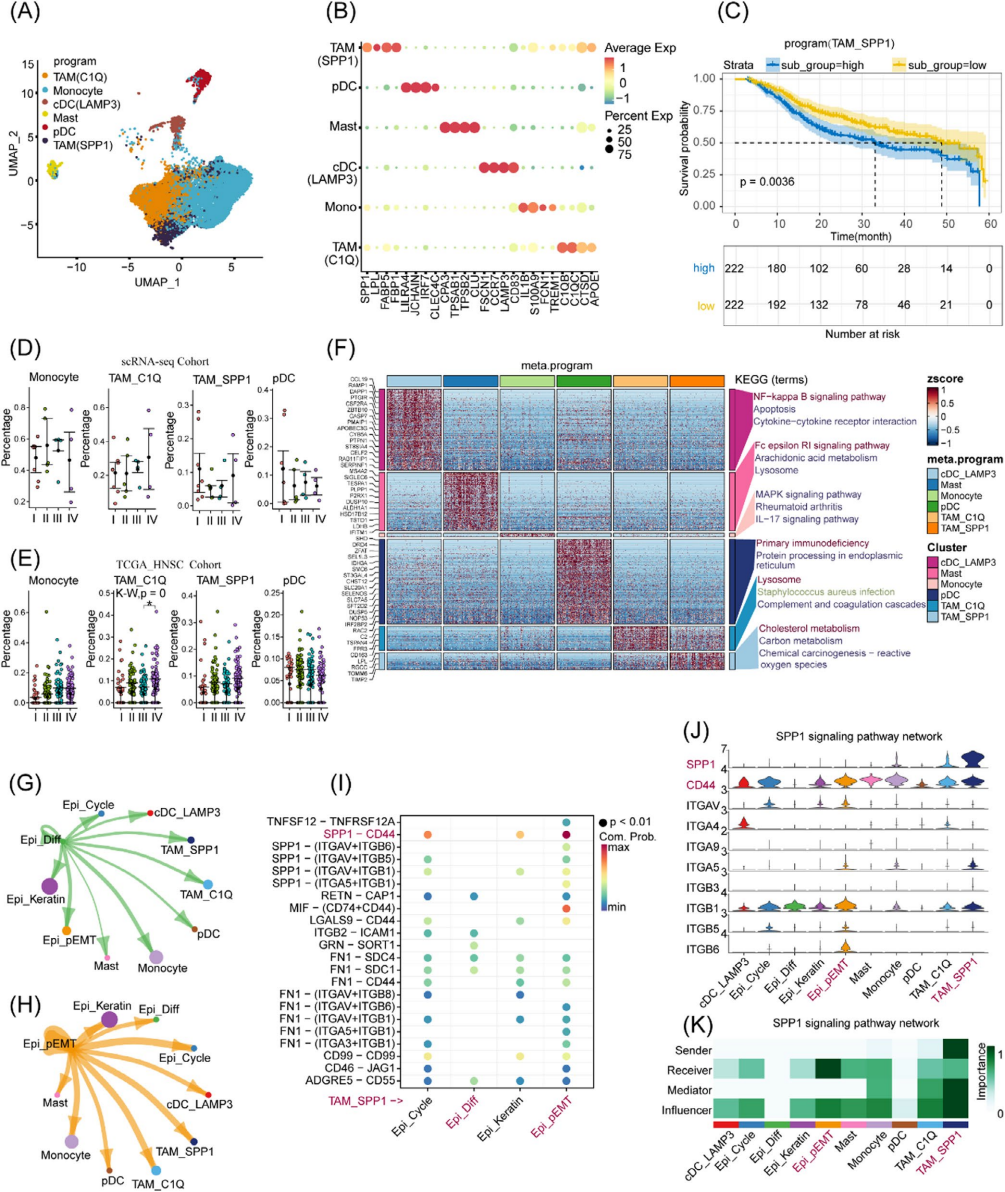
Investigation of myeloid cell–malignant cell state interactions. (A) UMAP plot delineates the cellular states within the tumour‐associated myeloid cell compartment. (B) A dot plot highlights the expression of myeloid cell‐specific markers. (C) Kaplan–Meier survival analyses in the TCGA_HNSC dataset highlight the prognostic significance of TAM(SPP1). (D, E) Dot plots the relative proportions of myeloid cells across single‐cell datasets and the TCGA_HNSC cohort. *p.adj value < 0.05 (Dunn's test with multiple comparisons). (E) Bar charts depict the enrichment analysis outcomes of differentially expressed genes specific to myeloid cells. (F) The interaction strength between Epi_EMT and myeloid cells is quantified. (G, H) Inferred ligand‐receptor interaction strength between Epi‐Diff or Epi_pEMT cells and myeloid cells, respectively. (I) The bubble plot of the ligand‐receptor pairs where TAM‐SPP1 acts as a sender to malignant cells. (J) The violin plot of the expression levels of ligand‐receptor genes across cell types in the SPP1 signalling network. (K) Highlighting the importance of each cell type in the SPP1 signalling.

In this study, we extracted myeloid cells for reclustering. Through the differentially expressed genes across distinct cellular clusters, we delineated seven unique myeloid cell subpopulations. Advancing our analysis, we derived six robust metaprograms from these cases, employing the cNMF approach to further elucidate cell state variability. As shown in Figure 5A,B and Figure S8B,E,H.

A compelling discovery in our study was the identification of two distinct cellular states of tumour‐associated macrophages (TAMs) with markedly divergent molecular signatures. TAM(C1Q) cells, characterised by robust expression of C1Q and APOE and observed consistently across multiple samples, were significantly enriched in lysosomal pathways, Staphylococcus infection pathways, and complement/coagulation cascades. In contrast, TAM(SPP1) cells, defined by elevated levels of SPP1 and FABP5, showed prominent enrichment in cholesterol and carbon metabolism pathways (Figure 5F), suggesting that these metabolic differences underpin distinct functional roles within the TME. Moreover, plasmacytoid dendritic cells (pDCs), marked by LILRA4 and CLEC4C expression, were enriched in primary immunodeficiency signalling pathways, whereas conventional dendritic cells (cDCs), defined by LAMP3 and FSCN1, exhibited enrichment in the NF‐κB signalling pathway. Additionally, mast cells (CPA3/TPSAB1) were enriched in the FcεRI signalling pathway, and monocytes (IL1B, S100A9) were significantly enriched in MAPK and IL‐17 signalling pathways.

Interestingly, through deconvolution analysis of the TCGA_HNSC dataset, we scrutinised the distribution of these programs across various clinical stages and their correlation with patient prognosis. Our results indicated a decreasing trend in the proportion of plasmacytoid dendritic cell (pDC) programs as tumours progressed, while the proportion of monocytic programs increased, aligning with trends observed in single‐cell samples (Figure 5D,E). Although not statistically significant, both TAM(C1Q) and TAM(SPP1) exhibited an increasing trend with tumour progression. In line with previous studies, prognostic analysis revealed that TAM(SPP1) programs were associated with poorer overall survival (Figure 5C), underscoring the clinical relevance of our findings [35, 36]. Subsequently, we shifted our focus to the cellular communication between Epi_Diff and Epi_pEMT states with myeloid cells.

Our results demonstrated that Epi_pEMT exhibited more pronounced interactions with TAM(SPP1) across multiple ligand‐receptor pairs (Figure 5G,H). Notably, within the SPP1 signalling pathway network, TAM(SPP1) emerged as a major secretor of the SPP1 ligand, with CD44 serving as the primary receptor on Epi_pEMT cells, highlighting a particularly prominent interaction (Figure 5I,J). The absence of Epi_Diff in this signalling network further underscores the specificity of this communication (Figure 5K).

### 3.6 Epi_Diff Exhibits Distinctive Interaction Features With NK/T Cells

We delineated the unique interaction profiles of Epi_Diff with NK/T cells, which are pivotal in tumour immunosurveillance. Epi_Diff represents a conserved expression signature with discernible biological roles. Given the cardinal importance of NK/T cells in the immunologic surveillance against cancer, we probed the nature of their interaction with malignant programmes, whether direct or indirect, and the distinctiveness of these patterns.

**FIGURE 6:**
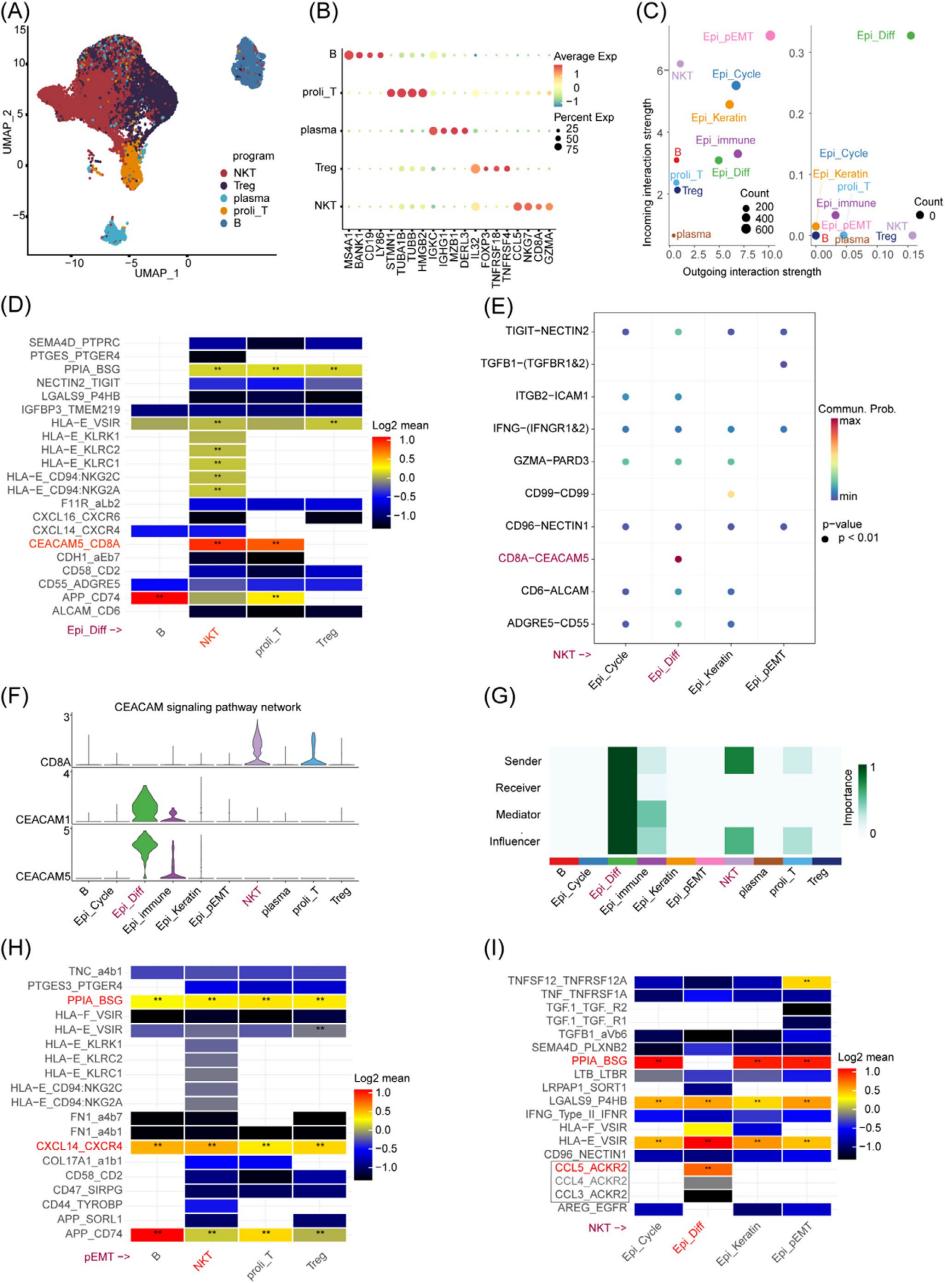
Analysis of the interaction profiles between malignant epithelial cells and NK/T cells. (A) UMAP plot delineates the lymphoid compartment, showcasing distinct cellular populations. (B) Dot plots highlight the signature genes that characterise various cell types within the lymphoid lineage. (C) Dotplots compare the intensity of signalling interactions between malignant cells and lymphoid cells, indicating the relative strengths of signal reception and emission. (D) Heatmap derived from CellphoneDB illustrates the interaction strength between Epi_Diff cells and lymphocytes. (E)The bubble plot from CellChat delineates the interaction characteristics between NK/T cells and malignant epithelial cells. (F, G) Violin plots and heatmaps present the enrichment of the CEACAM signalling pathway, highlighting its significance in cellular communication. (H) Heatmap from CellphoneDB displays the interaction profiles between malignant cells and NK/T cells. (I) Heatmap portrays the interaction features between NK/T cells and malignant epithelial cells from CellphoneDB. **Represent the top ligand‐receptor pairs that exhibit mean > 0 and *p*‐values less than 0.01.

Our analysis encompassed 56,459 lymphoid cells, revealing eight distinct subpopulations through differential gene expression profiling. cNMF analysis across the patient cohort identified five expression programmes, intriguingly devoid of signatures associated with T cell exhaustion (Figure 6A,B and Figure S8C,F,I).

We prioritised ligand‐receptor pairs based on their interaction affinity, with the top 50 pairs showcasing significant engagement. Notably, the CEACAM5‐CD8A and CCL5‐ACKR2 pathways emerged as pivotal in mediating interactions between Epi_Diff and NK/T cells. Within the CEACAM5‐CD8A axis, Epi_Diff cells functioned as principal signal emitters, with NK/T cells serving as primary responders, exhibiting robust interaction strength (Figure 6C–G and Tables S4 and S5). This interaction was corroborated on both CellChat and CellphoneDB platforms. Interestingly, CellphoneDB analysis also highlighted a substantial interaction via the CCL5‐ACKR2 pathway (Figure 6I). Typically, CCL5 is secreted to enhance immune cell recruitment; however, Epi_Diff cells expressed the antagonistic receptor ACKR2, suggesting a potential mechanism by which Epi_Diff may undermine immune cell infiltration and directly convey immunosuppressive signals, thereby dampening the antitumour efficacy of NK/T cells through a dual‐modality approach.

Conversely, the Epi_pEMT program, while demonstra ting significant interaction strength with NK/T cells through PPIA‐BSG and CXCL14‐CXCR4 pathways (Figure 6H), engaged in more intense and frequent interactions with mCAF1 and TAM(SPP1).

### 3.7 Immune Cell Interactions and Their Correlation With Epi_Diff and Epi_EMT

**FIGURE 7:**
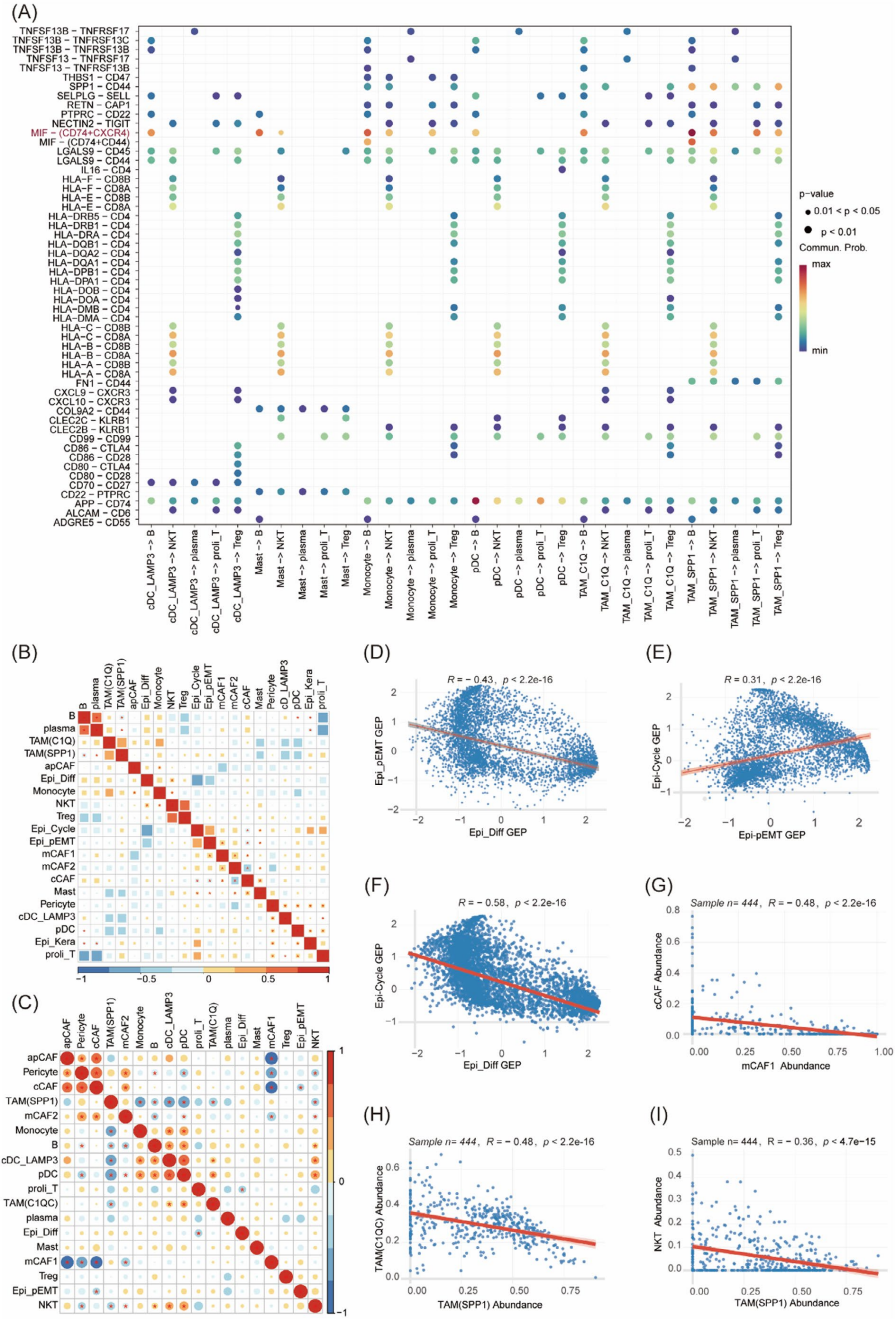
Interactions and correlations among immune and stromal cell subsets in HNSC. (A) Bubble plot showing communication probability of ligand‐receptor interactions from myeloid to lymphoid cells in HNSC (myeloid –> lymphoid). (B) Correlation heatmap of gene expression matrices across cell types from scRNA‐seq. (C) Correlation heatmap shows associations between malignant MPs scores and CAF/immune cell abundances (derived using CIBERSORTx) in TCGA‐HNSC samples. *p value < 0.05 (Spearman's rank correlation test). (D‐F) Scatter plots of MPs correlation in HNSC based on scRNA‐seq gene expression profiles (GEP). The Spearman correlation coefficient (R) and *p*‐value are displayed, with a trend line indicating the relationship. (D) Epi‐Diff vs Epi‐pEMT, (E) Epi‐pEMT vs Epi‐Cycle, (F) Epi‐Diff vs Epi‐Cycle. (G‐I) Scatter plots showing Spearman correlation analysis of CAF subtypes and immune cell abundances in TCGA‐HNSC: (G) mCAF1 vs cCAF, (H) TAM(SPP1) vs TAM(C1Q), (I) TAM(SPP1) vs NKT. *p value < 0.05 (Spearman's rank correlation test).

We analysed interactions among immune cell subsets. Key ligand‐receptor pairs included immune checkpoint molecules (CD80/86‐CTLA4) and HLA class I (HLA‐A/B/C) binding to TCR‐CD8A/B, suggesting concurrent antigen presentation and T cell exhaustion. TAM(SPP1) exhibited robust interactions with T cells via the MIF‐(CD74+CXCR4) axis (Figure 7A), potentially driving inflammation and immunosuppression in HNSC [37, 38].

Next, based on scRNA‐seq data analysis, we evaluated the correlation between malignant cell meta programs (Epi‐Diff, Epi‐pEMT and Epi‐Cycle) and TME cellular components at the gene expression profile level (Figure 7B). As anticipated: Epi‐Diff showed a significant negative correlation with Epi‐pEMT (*R* = −0.43, *p* = 2.2e‐16; Figure 7D); Epi‐pEMT was positively correlated with Epi‐Cycle (*R* = 0.31, *p* = 2.2e‐16; Figure 7E); Epi‐Diff exhibited a strong negative association with Epi‐Cycle (*R* = −0.58, *p* = 2.2e‐16; Figure 7F). Further integration of GSVA scores for malignant cell expression programs in TCGA‐HNSC samples with immune and stromal cell abundance inferred by CIBERSORTx revealed (Figure 7C): mCAF1 and TAM(SPP1) displayed significant negative correlations with their lineage‐originating cell populations (Figure 7G–H); TAM(SPP1) abundance inversely correlated with NK/T cell infiltration (*R* = −0.36, *p* = 4.7e‐15; Figure 7I).

## 4 Discussion

In this study, we employed the cNMF algorithm to robustly characterise cell types within both malignant and non‐malignant populations. The cNMF method offers distinct advantages in scRNA‐seq analyses by decomposing complex gene expression data into biologically interpretable meta‐programs, effectively capturing latent biological processes while mitigating technical noise and batch effect [12, 26, 39]. By integrating data from multiple patient samples across different anatomical sites and disease stages, we conducted an in‐depth analysis of the functional polarisation of malignant epithelial cells, stromal cells and immune cells in HNSC, as well as their cross‐compartment interactions. This work preliminarily revealed the dynamic evolutionary patterns within TME and their clinical relevance.

The Epi_Diff program, characterised by epithelial differentiation markers, may contribute to epithelial homeostasis maintenance via SPDEF‐mediated regulation [30, 40]. Furthermore, it demonstrates a correlation with favourable prognosis in the TCGA‐HNSC cohort. The significant negative correlations observed between Epi_Diff and both Epi_Cycle and Epi_pEMT (Figure 7D–F) suggest that this program may represent a relatively quiescent cellular state, which is consistent with its proposed role as a terminal differentiation state (Figure 3G). These findings align with recent studies suggesting that differentiated tumour cells may exhibit less aggressive phenotypes [13, 14, 41]. In contrast, the Epi_pEMT state exhibits partial epithelial–mesenchymal transition features but lacks classical EMT transcription factors, suggesting the existence of a non‐canonical invasion program driven by TEAD4‐mediated extracellular matrix remodelling [31]. This observation aligns with the established role of EMT in promoting invasiveness, metastatic potential and poorer prognosis [13, 42, 43, 44]. Furthermore, our findings align with this spatial paradigm [42], implying that these two cellular states may occupy distinct spatial niches within the tumour: Epi_Diff cells are likely enriched in the tumour core [42], exhibiting differentiated characteristics, whereas Epi_pEMT cells may localise to the tumour invasive front, displaying heightened invasiveness.

Specific malignant cell states demonstrated a preference for interacting with distinct cellular components of TME (Figure 4G–I). The Epi_pEMT state exhibited pronounced interactions with mCAF1 fibroblasts, particularly through the COL1A1–CD44 ligand‐receptor pair, which may promote tumour invasiveness by remodelling the extracellular matrix [45]. This observation suggests that combined targeting of TEAD4 signalling and CD44 inhibition could potentially disrupt the invasive‐stromal synergy. Similarly, the robust crosstalk between Epi_pEMT cells and TAM(SPP1) macrophages via the SPP1–CD44 axis implies a supportive role of myeloid cells in HNSC progression [33, 35]. The coordinated interactions between Epi_pEMT, TAM(SPP1) and mCAF1 hint at a co‐evolutionary relationship between malignant cell states and TME components, likely facilitating the formation of a pro‐metastatic niche.

Notably, the Epi_Diff program demonstrated selective interactions with natural killer (NK) and T cells via ligand‐receptor pairs including CEACAM5–CD8A and CCL5–ACKR2 (Figure 6D,E). These observations suggest that relatively quiescent tumour cells may modulate immune cells within the TME. The expression of atypical chemokine receptor 2 (ACKR2) by Epi_Diff cells points to a potential mechanism of immunemodulation. As a chemokine scavenger, ACKR2 may suppress immune cell infiltration by sequestering pro‐inflammatory chemokines [46, 47], which could contribute to the development of immune‐excluded tumour niches. However, our understanding of the molecular and cellular mechanisms of ACKR activity in HNSC remains poorly characterised. Emerging evidence in cervical cancer models indicates that ACKR2 promotes CD8+ T cell senescence and tumour recurrence [48]. These collective findings highlight ACKR2 as a potential therapeutic target [46, 49, 50]. Future studies should investigate whether targeting ACKR2 could mitigate its chemokine‐scavenging activity, potentially improving the response to immune checkpoint inhibitors.

Correlation analysis revealed inverse associations among cellular programs in the epithelial, stromal and immune compartments. Malignant epithelial cells exhibited mutually exclusive differentiation (Epi_Diff) and invasion (Epi_pEMT) programs, demonstrating a strong inverse correlation (Figure 7D). The Epi_Diff/pEMT ratio may serve as a potential indicator of tumour phenotypic plasticity, warranting further investigation. The positive association between Epi_pEMT and Epi_Cycle (Figure 7E) suggests a potential link to chemotherapy resistance. Pharmacological targeting of TEAD4 may attenuate the coordinated invasion –proliferation axis. In stromal cells, mCAF1 polarisation showed a strong negative correlation with cCAF abundance (Figure 7G), suggesting its role in maintaining invasive niches via extracellular matrix remodelling. TAM(SPP1) correlated inversely with TAM(C1Q) and NKT cell infiltration (Figure 7H,I), implicating their role in shaping an immune‐suppressive niche.

These findings propose a hierarchical ecosystem shaped by coordinated polarisation and inter‐compartment crosstalk, wherein three interdependent axes collectively contribute to tumour organisation: Differentiation‐invasion dichotomy (mutually exclusive Epi_Diff and Epi_pEMT states); Stromal reprogramming (imbalance between mCAF1 and cCAF populations); Immune lineage competition (inverse correlation of SPP1+ TAMs with NK/T). This multi‐scale architecture aligns with emerging concepts of tumour ecological hierarchy [17].

Although our study provides novel insights into the tumour ecosystems of HNSC, the limited sample size and absence of functional validation remain important limitations. While these findings advance our understanding of cellular heterogeneity and intercellular communication in HNSC, the cohort size may constrain broader applicability. Subsequent multi‐centre studies incorporating mechanistic experiments will be essential to validate these network interactions.

## 5 Conclusion

Our study demonstrates the potential of cNMF in resolving cellular heterogeneity and uncovering conserved meta‐programs that define both malignant and non‐malignant cell states in HNSC. These findings shed light on the functional interactions and polarisation phenomena within the epithelial, stromal and immune compartments, suggesting that tumour progression may result from the dynamic equilibrium of negatively correlated lineage‐specific populations. Moving forward, therapeutic strategies may benefit from an ecosystem remodelling perspective, potentially driving a paradigm shift in the treatment of HNSC.

## Author Contributions

**Donghui Jiang:** conceptualization (equal), data curation (equal), formal analysis (equal), funding acquisition (equal), methodology (equal), software (lead), supervision (supporting), visualization (equal), writing – original draft (equal), writing – review and editing (equal). **Xiaoguang Wu:** data curation (equal), formal analysis (equal), investigation (equal). **Yuanyuan Deng:** conceptualization (equal), methodology (equal), resources (equal), visualization (equal). **Xi Yang:** funding acquisition (equal), methodology (equal), software (equal). **Zhiqiang Wang:** conceptualization (equal), project administration (equal), resources (equal), software (supporting), supervision (equal). **Yong Tang:** conceptualization (equal), project administration (equal), supervision (equal). **Li He:** conceptualization (equal), project administration (equal), resources (equal), supervision (equal). **Xiaoguang He:** conceptualization (equal), project administration (equal), resources (equal), visualization (equal), writing – original draft (equal), writing – review and editing (equal).

## Conflicts of Interest

The authors declare no conflicts of interest.

## Supporting information


Figures S1–S8.

Table S1.

Table S2.

Table S3.

Table S4.

Table S5.

## Data Availability

The raw sequence data reported in this paper have been deposited in the Genome Sequence Archive [51] in National Genomics Data Center [52], China National Center for Bioinformation/Beijing Institute of Genomics, Chinese Academy of Sciences (HRA009256) that are publicly accessible at: https://ngdc.cncb.ac.cn/gsa‐human. The datasets used for integrated analysis in this study are available in the [GSE164690] repository, with case details provided in the original publication. [https://www.ncbi.nlm.nih.gov/geo/query/acc.cgi?acc=GSE164690].
