# Supplementary material for: Single‐Cell Profiling Reveals Conserved Differentiation and Partial EMT Programs Orchestrating Ecosystem‐Level Antagonisms in Head and Neck Cancer
Source: J Cell Mol Med. 2025 May 3;29(9):e70575. doi: 10.1111/jcmm.70575 (PMC12049153; doi:10.1111/jcmm.70575)
Supplement: Supplementary file 1 — Figures S1–S8. [file JCMM-29-e70575-s001.docx]

**
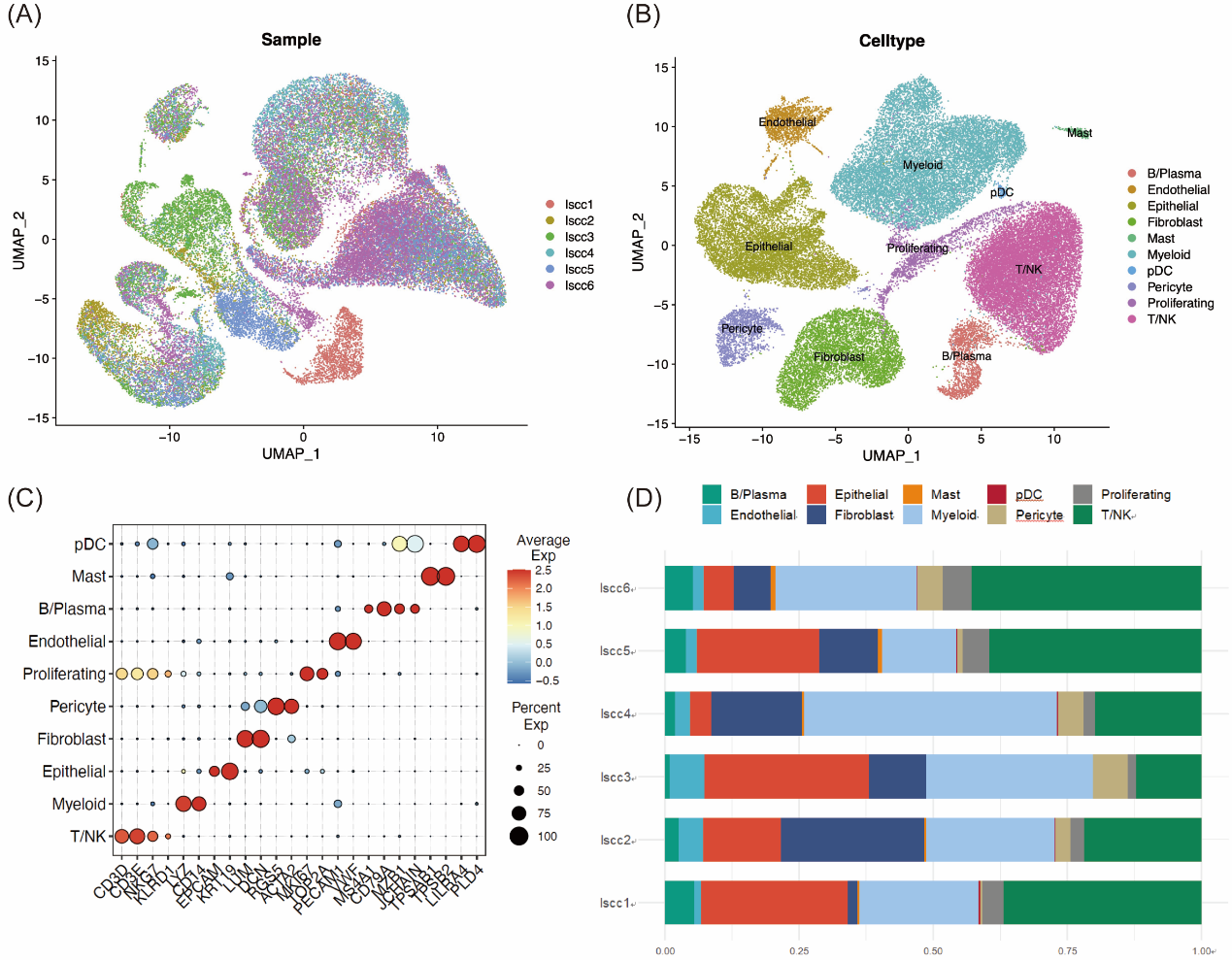
**

**Figure S1. scRNA-seq Analysis of Cellular Clustering and Identity Annotation in Six Laryngeal Cancer Samples. (A)** UMAP plot showing the distribution of cell clusters across six laryngeal cancer samples. **(B)** UMAP plot illustrating cell type clustering within the six samples. **(C)** Dot plot presenting differentially expressed genes across identified clusters. **(D)** Bar graph depicting the proportional distribution of annotated cell types across the six tumor samples.


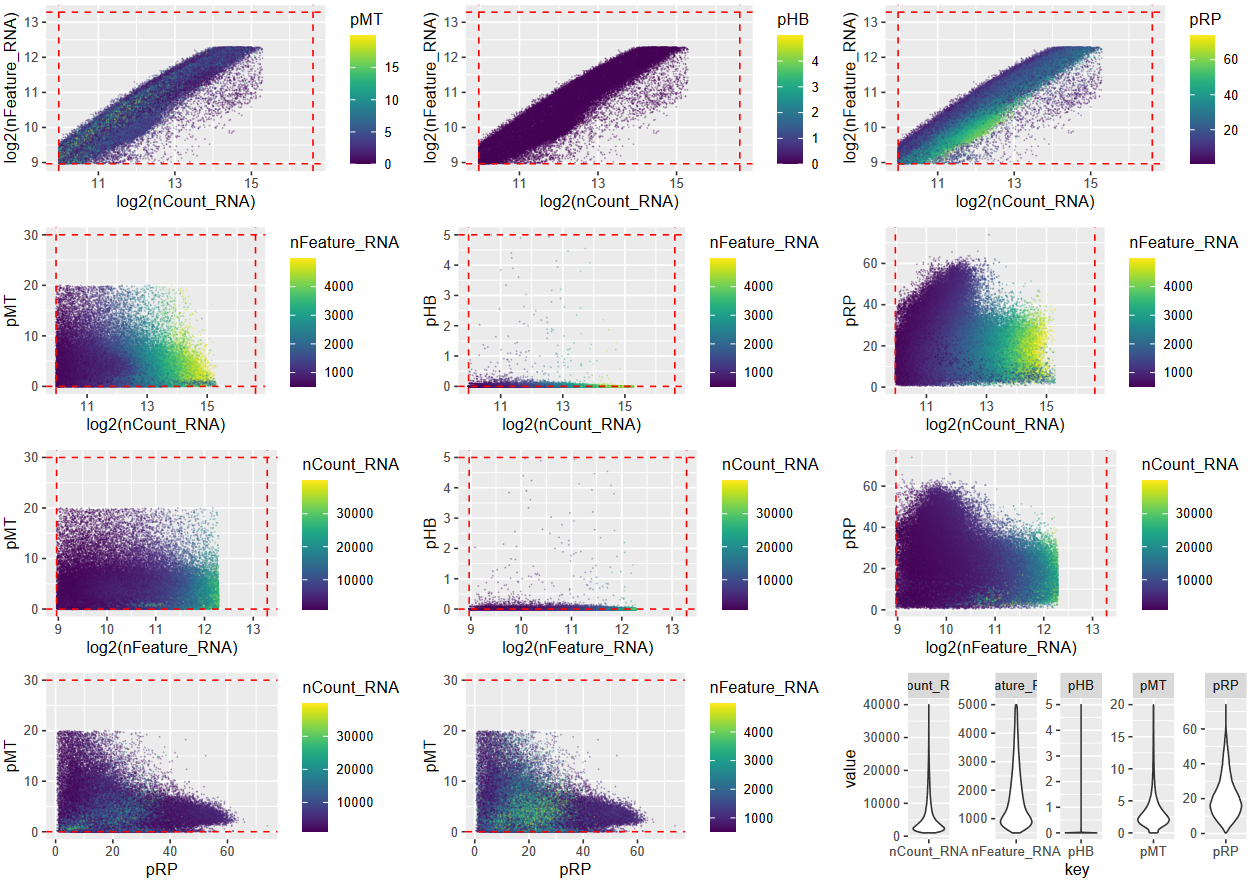


**Figure S2 Comprehensive Quality Control Enhances Single-Cell RNA-Seq Data from six laryngeal cancer samples integrated with GSE164690 Data.**

**Scatter plots** display log-transformed total RNA counts (nCount_RNA) vs log-transformed number of detected genes (nFeature_RNA) for individual cells. Cells are color-coded by the proportion of mitochondrial genes (pMT), hemoglobin genes (pHB), or ribosomal genes (pRP), as indicated. Red dashed lines represent quality control thresholds for RNA counts and gene numbers; cells outside these thresholds were excluded from further analysis.

**Violin plots** showing the distributions of key quality control metrics—including RNA counts (nCount_RNA), gene numbers (nFeature_RNA), pMT, pHB, and pRP—across all cells in the integrated dataset.


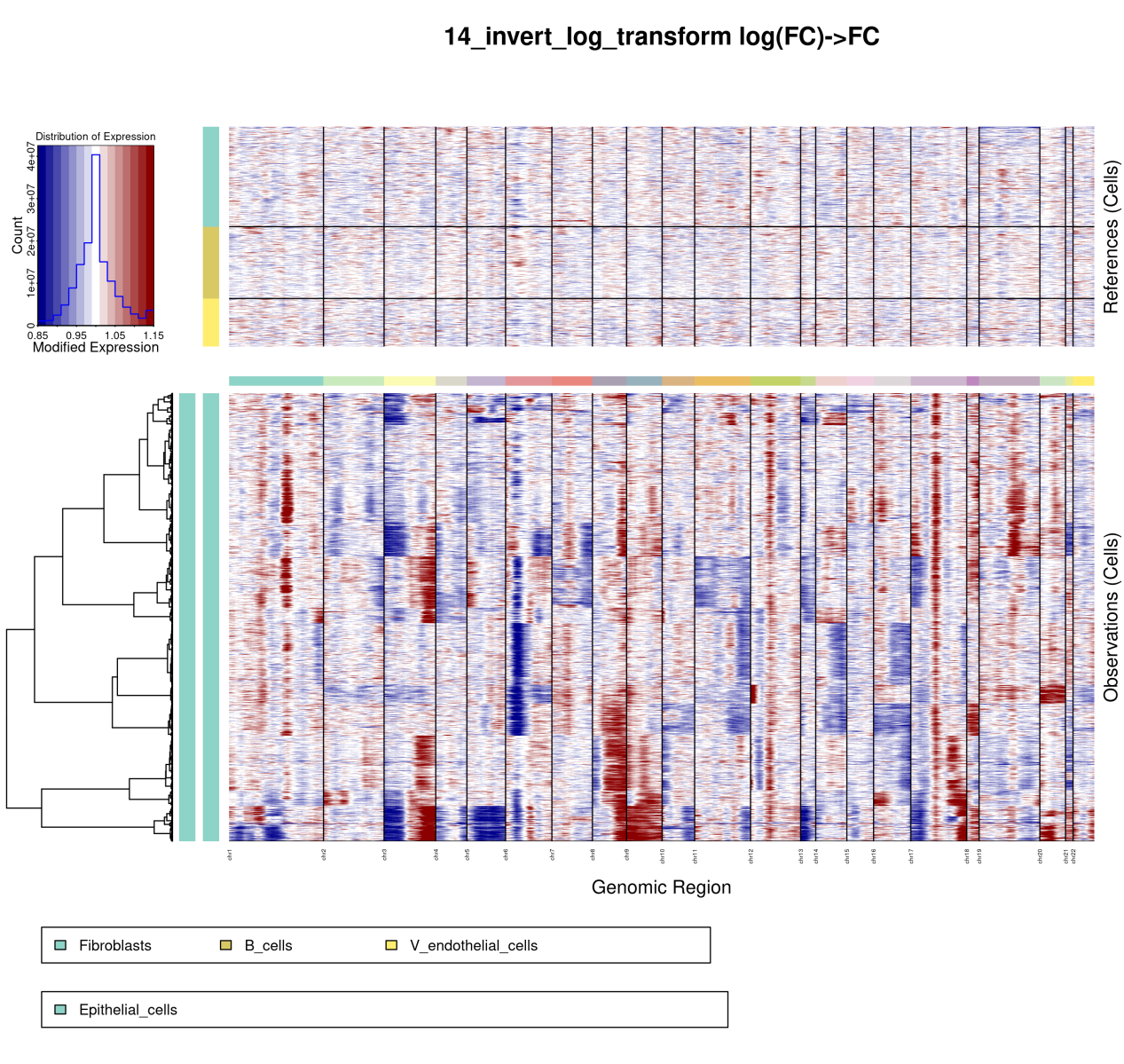
**
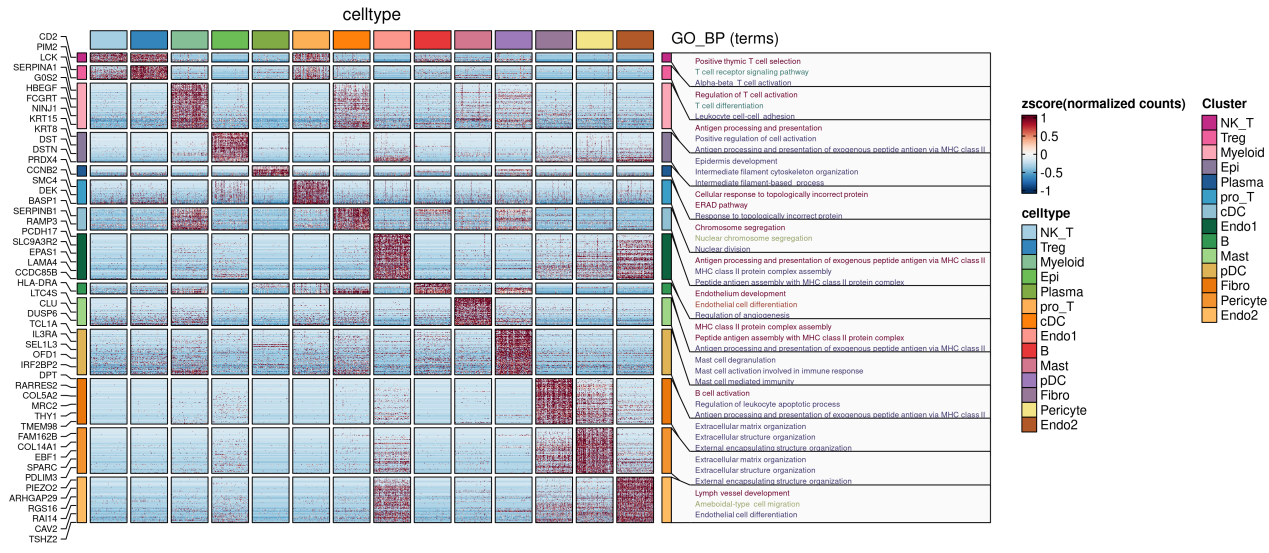
****Figure S3** **GO biological process enrichment heatmap highlights distinct functional signatures across cell types.** Columns represent cell types (e.g., NK_T, Treg, Myeloid, Epi), and rows correspond to differentially expressed genes (DEGs). GO terms enriched in each cell population are shown on the right. Heatmap colors reflect z-scores, with red indicating upregulation, blue downregulation, and white for no change.

**Figure S4. InferCNV analysis of epithelial-like tumor cells, comparing copy number variation (CNV) profiles to reference cells.** The left density plot shows the distribution of expression levels across cells, with deviations indicating potential CNVs. The central heatmap displays CNV patterns, with each row as a cell: "Reference (Cells)" above and "Observations (Cells)" below. Red and blue gradients denote amplifications and deletions, respectively. Cell types (fibroblasts, B cells, vascular endothelial cells, epithelial cells) are color-coded on the left, and the x-axis shows genomic regions segmented by chromosome.

**
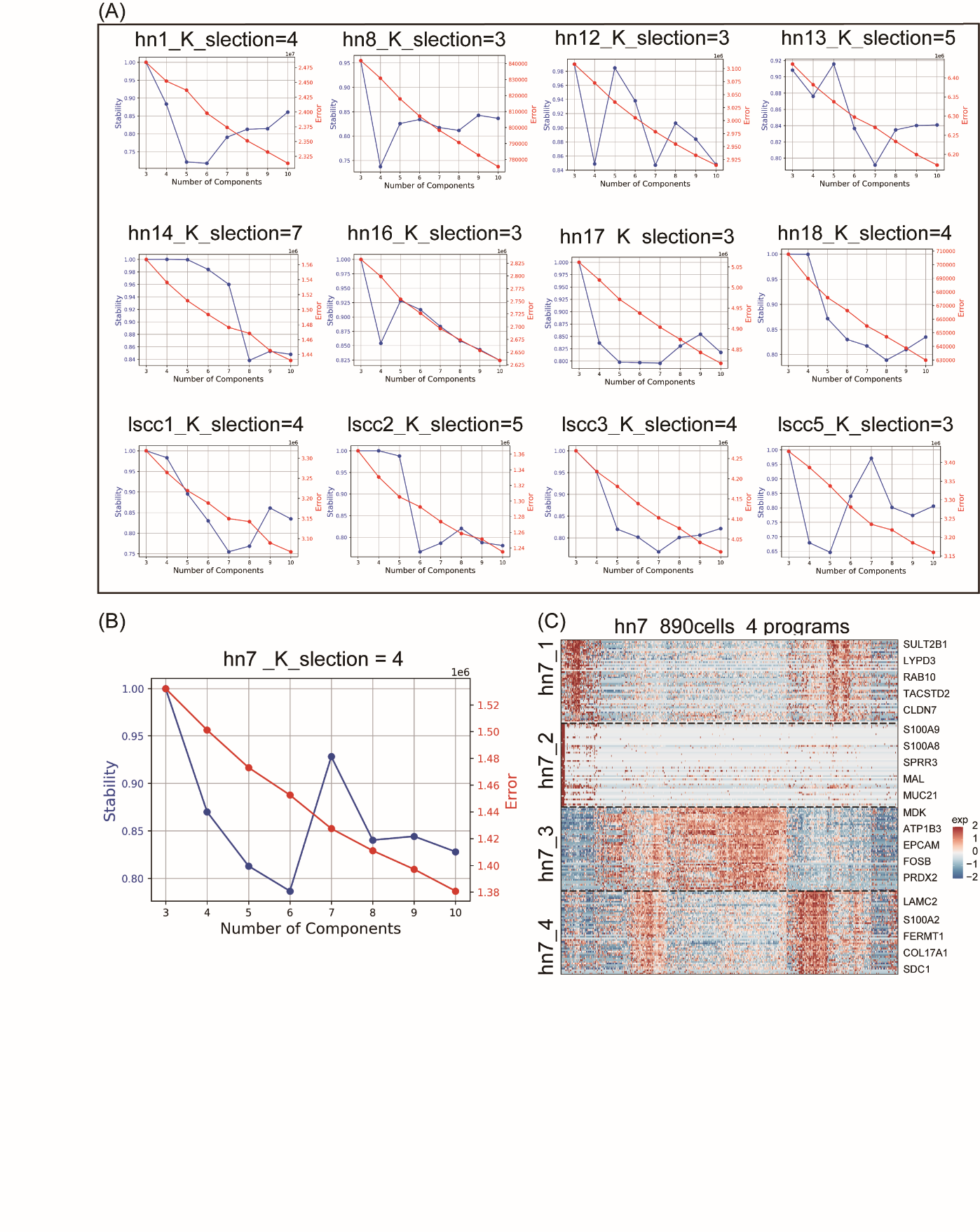
**

**Figure S5. cNMF Analysis of K-value Selection and Expression Program Decomposition in hn7 Sample. (A)** The optimal K value for each sample was chosen based on model stability and reconstruction error. Stability reflects the consistency of decomposition results across different initializations, while reconstruction error indicates the model’s fit to the original data. **(B)** In sample hn7, K=4 provided an ideal balance, achieving high stability and an acceptable reconstruction error, thereby ensuring model simplicity while retaining biological relevance. **(C)** The heatmap illustrates four distinct expression programs identified in 890 malignant cells from hn7, with the top five genes associated with each program listed on the right.


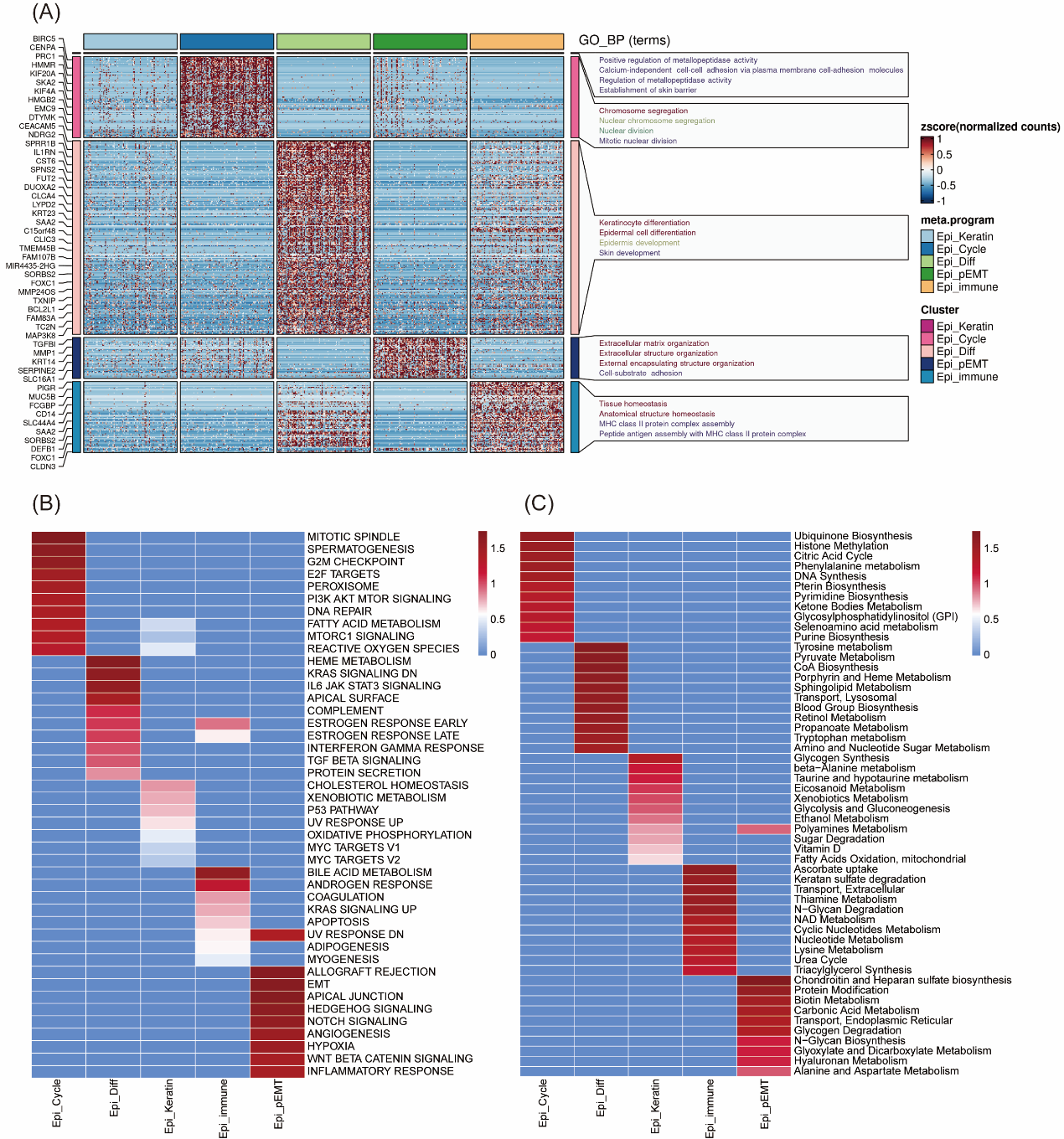


**Figure S6. Functional Enrichment Analysis of Expression Programs in Malignant Epithelial Cells. (A)** GO Biological Process (GO_BP) analysis heatmap showing enriched biological processes associated with each expression program. **(B)** Hallmark gene analysis heatmap highlighting pathway-level features for each program. **(C)** Metabolic gene set analysis heatmap indicating enrichment of metabolic pathways across expression programs.

**
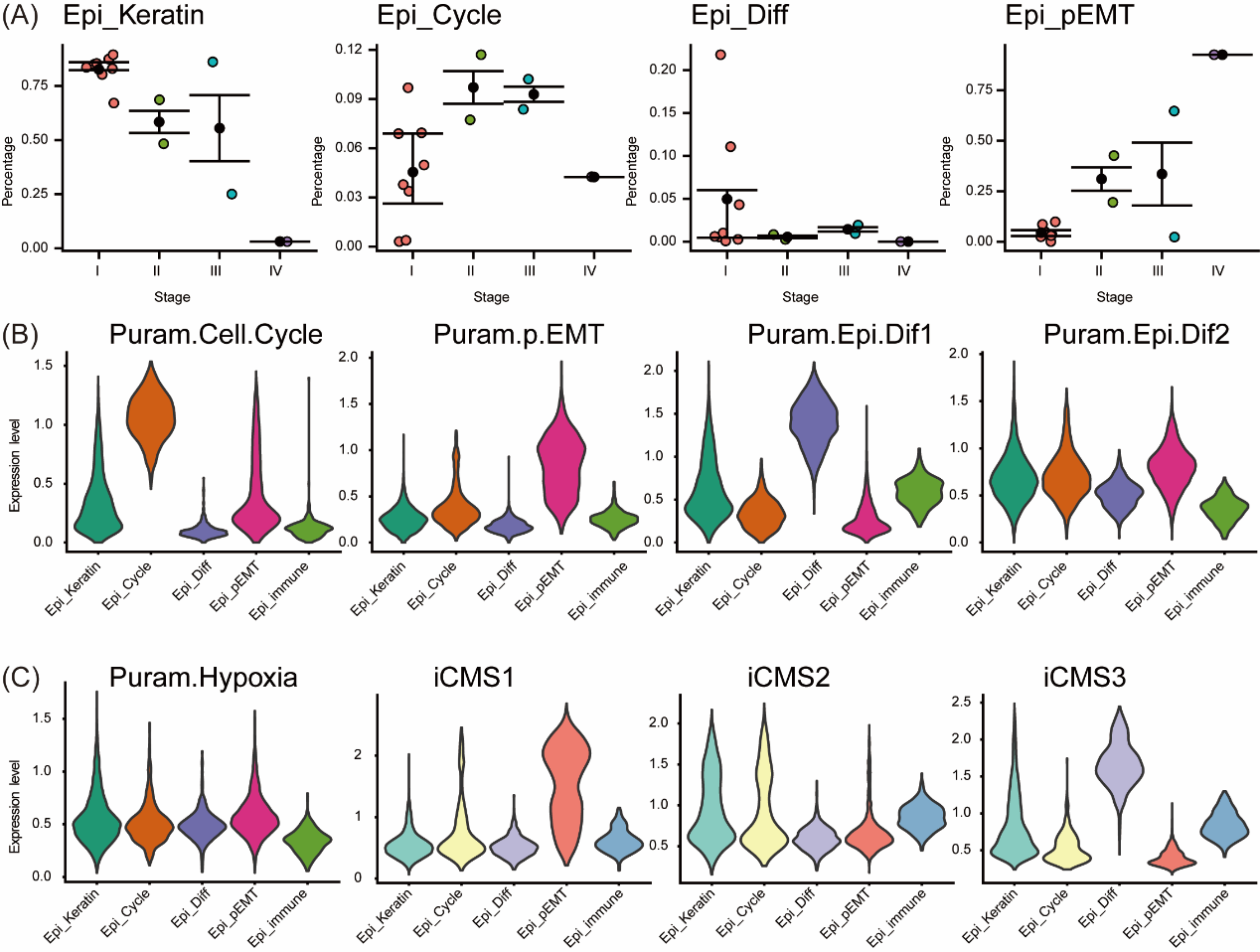
**

**Figure S7. Core Expression Program Dynamics and Comparative Analysis in Malignant Epithelial Cells across Tumor Stages. (A)** Dot plot depicting the distribution of core expression programs (Epi_Keratin, Epi_Cycle, Epi_Diff, Epi_pEMT) across tumor stages I–IV, demonstrating shifts in program prevalence with tumor progression. **(B-C)** Violin plots comparing scores of malignant expression programs defined in this study with cellular phenotypes reported in prior studies.


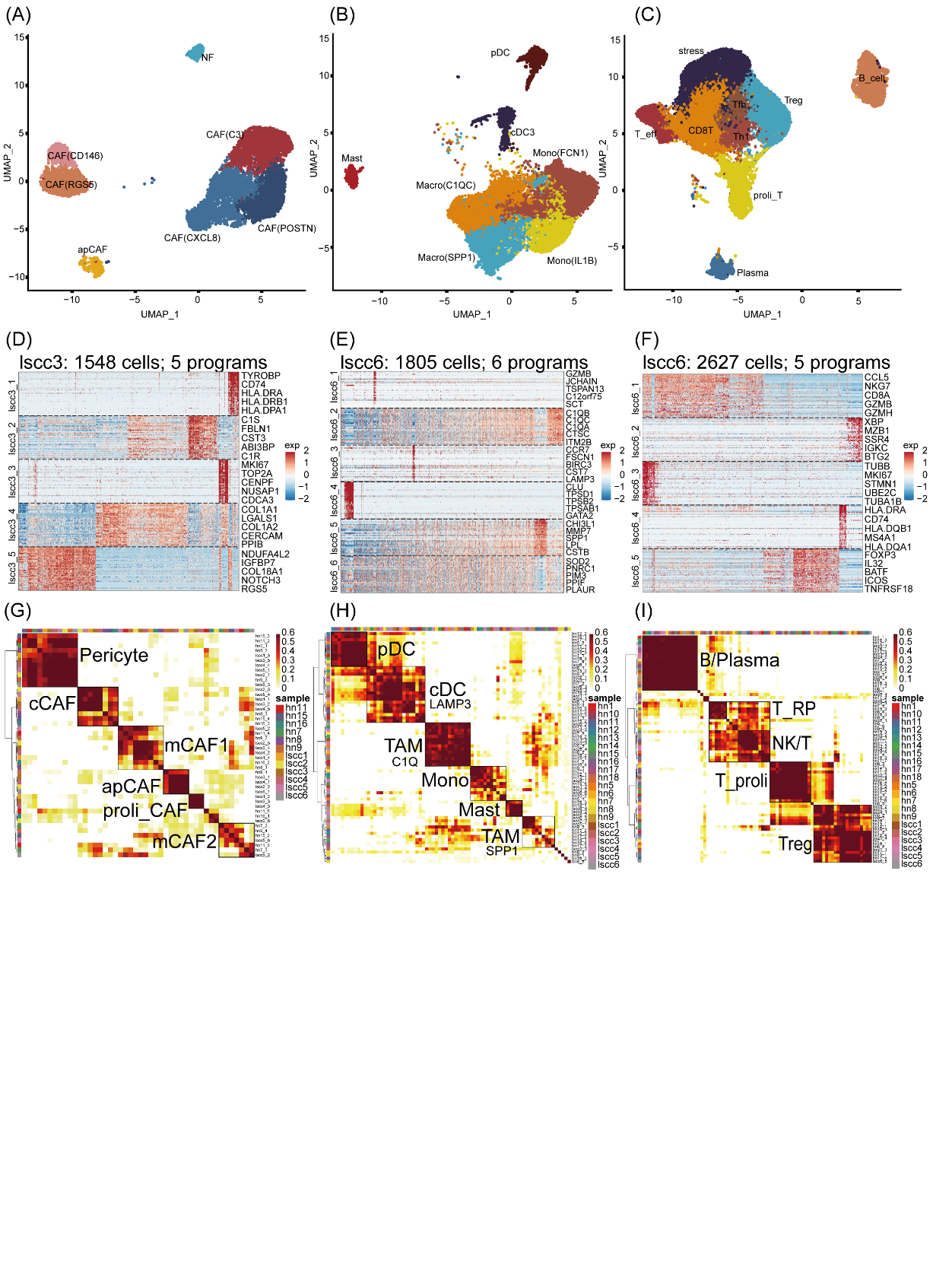


**Figure S8.** **Decomposition of Non-Malignant Cell Expression Programs in the Tumor Microenvironment via cNMF. (A-C)** UMAP plots displaying cell clustering of CAFs, myeloid, and lymphoid cells, respectively. **(D-F)** Expression program decomposition in representative samples, with the top five genes for each program listed. **(G-I)** Heatmaps showing hierarchical clustering of expression programs in CAFs, myeloid, and lymphoid cells. Clusters enclosed in boxes represent metaprograms, indicating expression programs shared across multiple samples.
